# Myopia Genetics and Heredity

**DOI:** 10.3390/children9030382

**Published:** 2022-03-09

**Authors:** Yu-Meng Wang, Shi-Yao Lu, Xiu-Juan Zhang, Li-Jia Chen, Chi-Pui Pang, Jason C. Yam

**Affiliations:** 1 Department of Ophthalmology and Visual Sciences, The Chinese University of Hong Kong, Hong Kong SAR, China; ymwang917@gmail.com (Y.-M.W.); shiyaolu@link.cuhk.edu.hk (S.-Y.L.); zhangxiujuan@cuhk.edu.hk (X.-J.Z.); lijia_chen@cuhk.edu.hk (L.-J.C.); cppang@cuhk.edu.hk (C.-P.P.); 2 Department of Ophthalmology, Guangzhou First People’s Hospital, School of Medicine, South China University of Technology, Guangzhou 510006, China; 3 Hong Kong Eye Hospital, Kowloon, Hong Kong SAR, China

**Keywords:** myopia, genes, ethnicity, environment, education, time outdoors, atropine

## Abstract

Myopia is the most common eye condition leading to visual impairment and is greatly influenced by genetics. Over the last two decades, more than 400 associated gene loci have been mapped for myopia and refractive errors via family linkage analyses, candidate gene studies, genome-wide association studies (GWAS), and next-generation sequencing (NGS). Lifestyle factors, such as excessive near work and short outdoor time, are the primary external factors affecting myopia onset and progression. Notably, besides becoming a global health issue, myopia is more prevalent and severe among East Asians than among Caucasians, especially individuals of Chinese, Japanese, and Korean ancestry. Myopia, especially high myopia, can be serious in consequences. The etiology of high myopia is complex. Prediction for progression of myopia to high myopia can help with prevention and early interventions. Prediction models are thus warranted for risk stratification. There have been vigorous investigations on molecular genetics and lifestyle factors to establish polygenic risk estimations for myopia. However, genes causing myopia have to be identified in order to shed light on pathogenesis and pathway mechanisms. This report aims to examine current evidence regarding (1) the genetic architecture of myopia; (2) currently associated myopia loci identified from the OMIM database, genetic association studies, and NGS studies; (3) gene-environment interactions; and (4) the prediction of myopia via polygenic risk scores (PRSs). The report also discusses various perspectives on myopia genetics and heredity.

## 1. Introduction

Myopia is the most common cause of vision loss, with an uncorrected refractive error being the leading ocular disorder, causing visual impairment worldwide [1]. A refractive error reaching −6.00 diopters (D) or an axial length (AL) exceeding 26 mm is defined as high myopia, which increases susceptibility to ocular complications [2]. Now a global public health burden [3,4], myopia has become significantly more prevalent across East Asia [1,5]. In particular, the prevalence of myopia among young adults in the urban areas of East Asian countries has risen to 80–90% [6,7], whereas the same trend has been observed in other areas [1,5]. Given current trends, the global population affected by myopia is estimated to increase significantly by the year 2050, among which 10% will develop high myopia [8]. Axial length elongation is irreversible once it occurs [9,10]. Since the pathology of myopia has no cure, the development of myopia must be controlled or prevented to avoid serious ophthalmic consequences.

The etiology of myopia is heterogeneous and multifactorial, being affected by various environmental and genetic factors [1]. Epidemiological studies indicate that increased outdoor activities and reduced work play important roles in controlling myopia incidence [11]. On the other hand, the role of genetics varies across ethnic groups. Heritability estimates reported from several studies on twins, including Asian and Caucasian populations, ranged from 50% to 90% [12,13,14,15,16,17]. However, the results based on the twin study could represent fully the role of genetic effects. Twin study designs have more power to detect heritable effects in variance component models of myopia, but family studies have more power to detect shared environmental effects [16]. Another major argument for the role of genetic and environmental factors in myopia development is that myopia prevalence keeps increasing rapidly in the past decades; however, the gene pool in humans remains relatively stable across centuries. Therefore, genetic variations cannot fully explain the majority of variations in myopia risk. Environmental factors have been considered as a major factor for myopia, especially in school children, while genetic factors account for a small proportion [18]. In addition, the known genetic loci from genetic exploration in the recent 20 years, only explained less than 20% of the heritability for myopia in European and even less in East Asian children [19]. Another earlier single nucleotide polymorphisms (SNP)-based heritability estimation showed a heritability range from 25% to 35% [20]. This might imply technical limitations in genetic association discovery.

Nevertheless, gene investigations are still important for improving the understanding of gene components relative to the disease onset/susceptibility and the molecular mechanisms of impaired vision [19,21]. This review summarizes the major findings on myopia genetics and heredity.

## 2. Genetic Architecture of Myopia

### 2.1. Prevalence of Myopia

The reported prevalence of myopia varies across populations [8]. It has been reported as 30.6% among Caucasians across Europe [22], 41.6% among a population of both white and black Americans in the United States [23], 41.8% among an elderly Japanese population, and 90% among high school students in East Asia [1]. These population-based epidemiological investigations have shown the essential impacts of genetic factors on the onset and progression of myopia. Moreover, a history of myopia among parents (“parental myopia”) significantly correlates with the myopia occurrence of their children [24].

### 2.2. Myopia-Related Axial Length Elongation and Its Effects on the Fundus

During the neonatal period, refraction is essentially hyperopic [25]. The acceleration of axial eye growth predominantly occurs from school years onwards and tends to stabilize in early adulthood [26]. Excessive elongation of axial length leads to myopia. Longer axial lengths have been found among myopic children with a myopic family history [27]. Studies among different populations, including Caucasians, Koreans, and Chinese, have also observed that AL has higher heritability coefficients (range: 0.67–0.94) compared to other refractive errors (0.58–0.88) [17,28,29]. In particular, a study on Chinese children with myopic parents discovered rapid eye growth having occurred even before the onset of myopia [30]. Lastly, with high myopia, the fundus is affected by disorders, including optic disc tilt, fundus pallor, and tessellation, and peripapillary atrophy, which lead to major pathological manifestations [31].

## 3. Identification of Myopia Loci

### 3.1. Online Mendelian Inheritance in Man (OMIM) Database

At present, the OMIM database has identified 26 myopia loci from linkage analyses in myopia pedigrees (Table 1). These loci are scattered across all autosomal chromosomes and the X-chromosome. Most show autosomal dominant inheritance, whereas *MYP18* and *MYP23* were discovered among families with autosomal recessive myopia. Among all reported loci, only seven candidate genes and mutations have been replicated thus far. These loci have stronger genetic effects and trends to affect disease onset with Mendelian inheritance. In general, mutations at these loci are rare but with large pathogenetic effects, whereas variants associated with common forms of myopia have higher allelic frequency and smaller effects.

#### 3.1.1. Genetic Loci for Myopia among Asian Cohorts

*MYP16*, an autosomal dominant locus located on chromosome 5p15.33-p15.2, was first identified among Hong Kong Chinese subjects (Table 1) [16]. A later GWAS among Singaporean Chinese subjects found that two single nucleotide polymorphisms (SNPs), rs12716080 and rs6885224 in *CTNND2* near *MYP16*, showed significant associations with high myopia [57]. The significance of rs6885224 was replicated for the Japanese cohort in the same study [57], and for a Chinese cohort in Guangzhou, albeit with an opposite allelic effect for the latter [57,58]. Lastly, a multi-center collaborative study identified rs1021711 near *MYP16* as a susceptible SNP for myopia [59].

Turning to other loci, a second locus, *MYP18*, was mapped to chromosome 14q22.1-q24.2 [45]; however, no replication study for this locus has been reported. Unlike other loci, *MYP20* was identified from a GWAS that examined 493,947 SNPs among 419 highly myopic Chinese individuals and 669 unrelated controls. A number of SNPs in the *MIPEP*, *C1QTNF9B-AS1* and *C1QTNF9B* within this locus showed strong associations with high myopia [47].

A pedigree segregation analysis for autosomal dominant high myopia revealed homozygosity for a missense mutation of the *CCDC111* gene at the *MYP22* locus in the proband and with heterozygosity in all other affected family members [50]. A candidate gene study among Chinese subjects also identified a frameshift variant (p.V131Gfs*6) in *CCDC111* among highly myopic patients [60]. However, the same variant had been reported with no phenotypic information among ten East Asians and one Bengali in Bangladesh under Phase 3 of the 1000 Genomes (1000 G) Project [61]; its genetic significance thus remains unclear.

In the *SLC39A5* (*MYP24*) gene on chromosome 12q13, a heterozygous nonsense mutation (c.141C > G:p.Y47*) was identified [53]. Functional analysis indicates that *SLC39A5* plays a role in ocular elongation. Other *SLC39A5* mutations in highly myopic patients included one frameshift (p.Arg243fs*140), three missense variants (p.G413A, p.N167S, p.L445P and p.T527I), and one nonsense variant [60,62].

The final locus, *MYP25*, was discovered through whole-genome linkage analysis and exome sequencing in a three-generation Chinese pedigree [54]. Moreover, two mutations (p.D128N and p.184delH) were identified in highly myopic Chinese patients [60], while another mutation (p.K383E) was identified in moderate myopia, its pathogenicity being validated in vitro [63].

#### 3.1.2. Genetic Loci for Myopia among Caucasian Cohorts

It has been identified that the *MYP5* locus was located on chromosome 17q21-q22 for autosomal dominant high myopia [36]. Another study identified the *COL1A1* (Collagen Type I Alpha 1 Chain) gene, which then regulates collagen fibril assembly and interactions in multiple tissues [64]. *COL1A1* variants in this gene have been found for Stickler syndrome, a hereditary connective tissue disorder associated with high myopia [65]. 

Regarding other loci, a 2004 study involving 506 pairs of Caucasian twins used a genome-wide linkage scan to reveal linkage peaks at chromosomes 11p13 (MYP7), 3q26 (*MYP8*), 4q12 (*MYP9*), and 8p23 (*MYP10*) [38]. In 2005, the *MYP12* locus on chromosome 2q37.1 was identified with autosomal dominant high myopia [63]. However, the sequencing of the S-antigen (SAG) and the diacylglycerol kinase-delta (*DGKD*) gene using the positional candidate gene approach found no causative mutations for the locus [40]. Later, in 2006, the *MYP14* locus on chromosome 1p36 was identified [42]. Subsequently, *MYP15* on chromosome 10q21.1 was identified among a large Hutterite family in 2007 [43], and *MYP17* on chromosome 7p15 was identified through a non-parametric multipoint linkage analysis among 26 French families in 2008 [44]. Lastly, a study reported putative mutations of SCO2 in *MYP6* associated with autosomal dominant high myopia among white Americans [66]. Since then, no other candidate genes associated with myopia have been identified from the myopia loci reported among Caucasian families [66].

#### 3.1.3. Genetic Loci and Genes for Myopia Replicated across Multiple Ethnic Groups

In 1981, Bartsocas and Kastrantas reported X-linked inheritance in a Greek family affected by myopia, suggesting the existence of an X-linked form of myopia [67]. A locus for Bornholm eye disease (BED) was identified in the distal part at Xq28 (Table 1) [32]. X-linked recessive inheritance of the *MYP1* locus was also identified [68]. However, sequencing analysis of the coding and adjacent intronic regions did not show any mutations associated with myopia in *MYP1* [68]. In 2011, X-linked recessive inheritance was reported in two large multigenerational Indian pedigrees with nonsyndromic myopia (mean refractive error, −8.48 D; range, −6 to −23 D) [69]. Sequencing analysis revealed the LVAVA haplotype and a frameshift mutation in the OPN1LW gene located in *MYP1* [70]. Lastly, in 2017, Orosz et al. found co-segregations of the *LVAVA* and *MVAVA* haplotypes in *OPN1LW* and *OPN1MW*, associated with X-linked nonsyndromic high myopia among young patients in a three-generation Hungarian family [71]. The co-segregations were also associated with progressive cone-rod dystrophy featuring deuteranopia and protanopia, indicating the complexity of non-simplex high myopia [71].

In 1998, a genome-wide screening reported the investigation of eight families with high, early-onset, autosomal dominant myopia (<−6 D) [33]. After excluding candidate loci for syndromic myopia, a significant linkage was found on chromosome 18p [33]. Significant associations were also reported between SNPs in TGIF (transforming growth beta-induced factor) and high myopia among Chinese subjects [72]; however, these associations could not be replicated in another Chinese cohort [73] or in a Japanese cohort [74]. Moreover, no association between the TGIF variants and refraction or ocular biometric measures was reported among Caucasians, indicating that TGIF is unlikely to play a major role in myopia [75].

Young et al. reported a significant association of autosomal dominant high myopia to the *MYP3* locus (Table 1) [35]. In a Chinese cohort in Taiwan, an SNP in the lumican (*LUM*) protein on the 12q21.3-q22 gene was significantly higher presenting among myopia patients; it resulted in lower luciferase assay activity compared to the wild variety [76]. Scleral thinning is a feature of the development of high myopia. Scleral remodeling affecting the scleral extracellular matrix could occur during myopia progression due to axial length elongation [77]. Another candidate gene in the *MYP3* locus is IGF-1. The SNP rs6214 showed a positive association with both the high-grade and any-myopia groups (*p* = 2 × 10^−3^ for both groups) after corrections for multiple testing [78].

Among a cohort of 44 American families of Ashkenazi Jewish descent, linkage analysis was performed on chromosome 22, producing a dense map of >1500 SNP markers for fine mapping and association analyses; the study identified a refined genetic locus for myopia at *MYP6* on 22q12 (Table 1) [37]. This linkage was later replicated in the Beaver Dam Eye Study on Caucasians [79]. Moreover, in 2013, SCO2 (Synthesis of Cytochrome C Oxidase 2), a putative disease-causing gene in *MYP6*, was identified [66].

The study involving 506 Caucasian pairs of twins considered *PAX6* as a candidate gene for myopia, since it was the closest to the highest linkage peak [38]. The association of *PAX6* variants with susceptibility to high myopia was subsequently demonstrated [80]. The SNP rs662702 of *PAX6* affected the risk of extreme myopia [81]. Moreover, the risk allele could reduce PAX6 protein levels, which may be involved in the underlying mechanism of myopia pathogenesis [81]. Increases in the length of the *PAX6* P1 promoter AG dinucleotide repeatedly affect the transcription activity and are associated with high myopia [82].

A genome screening mapped the *MYP4* locus to chromosome 7q36 [83]. A subsequent study that surveyed more families and subjects in the same population performed multipoint parametric and non-parametric analyses to explore this locus. However, no significant findings were detected in the parametric model, even in the 7q36 region. Instead, significant linkages to another region, 7p15 (between markers *D7S2458* and *D7S2515*), were found from the non-parametric analysis of the population [44]. The Human Genome Organization (HUGO) later approved the replacement of *MYP4* with *MYP17* as the new locus (Table 1).

In summary, the loci derived from linkage analyses of pedigrees can seldom be repeated in later studies. This inability to replicate findings may be explained by the occurrence of rare forms of myopia or even rare syndromes, including myopic phenotypes. Moreover, the alleles related to myopia in genetic loci have low frequencies in the population and are thus less likely to be detected in population-based studies. As mentioned before, the rapidly increased rate of myopia in recent decades and earlier onset are strongly associated with schooling. Therefore, common myopia, known as school myopia, is most likely to have a different contribution pattern of genetic and environmental risk factors. The distinction between common and rare forms of myopia is important in the explorations of etiology and susceptibility of this disease. Flitcroft et al. (2018) studied genetic contributions in both forms of myopia. They conducted pathway analysis and identified overlapped biological processes between the common form and syndromic myopia which is associated with at least one other medical condition besides nearsightedness. According to their analyses, common variants in or near the loci identified from syndromic myopia are enriched for genetic profiles in the common form [84]. To discover the variants affecting common myopia in the population, genetic association approaches have been applied.

### 3.2. Genetic Association Studies for Myopia, Refractive Error, Axial Length, and Macular Thickness

GWAS and follow-up association studies have been conducted among different ethnic groups to discover the genetic factors for myopia. These studies have repeatedly found that many genetic variants are associated with phenotypic characteristics related to myopia and high myopia, including refractive errors, axial length, and macular thickness. 

#### 3.2.1. SNPs in Myopia-Related Genes from Association Studies

GWAS have associated myopia with various genes of known biological, physiological or functional relevance. Among them, association analysis of tag SNPs in *TGFB1* (transforming growth factor beta1) among Chinese high myopia and control subjects detected a significant association with myopia [85] (Table 2). Likewise, a Japanese study associated the rs577948 SNP at chromosome 11q24.1, between *BLID* and *LOC399959*, with high myopia at the GWAS-significant level [86]. Moreover, the *CHRM3* gene was reported to associate with susceptibility to high myopia among Chinese subjects, with a role in myopia pathogenesis [87]. Lastly, the rs6885224 SNP at the *CTNND2* gene on chromosome 5p15.2 was identified from a high myopia GWAS of several Asian datasets [57]. 

In 2011, a GWAS on a Han Chinese cohort revealed a strong association with myopia for rs10034228 [88]. Another GWAS for Chinese subjects mapped the *MYP20* locus to a region adjacent to the *MIPEP, C1QTNF9B-AS1*, and *C1QTNF9B* genes (Table 2) [47]. IGF-1 haplotypes associated with genetic susceptibility to high myopia were found [89]. 

**Table 2:** Genome-wide association studies in myopia and top findings.

First Author	Publication Date *	Phenotype	Discovery Stage		Replication Stage		Genes/Loci **
			Cases	Controls	Cases	Controls	
Nakanishi H. [86]	25 September 2009	High myopia	297 Japanese ancestry	934 Japanese ancestry	533 Japanese ancestry	977 Japanese ancestry	*BLID-LOC399959*
Li Y.J. [57]	20 November 2010	High myopia	65 children and 222 adults of Singaporean Chinese	238 children and 435 adults of Singaporean Chinese	959 Japanese ancestry	2128 Japanese ancestry	*CTNND2*
Li Z. [88]	19 April 2011	High myopia	102 Chinese ancestry	335 Chinese ancestry	2891 Chinese ancestry	10,071 Chinese ancestry	*4q25*
Shi Y. [47]	2 June 2011	High myopia	419 Chinese ancestry	669 Chinese ancestry	2803 Chinese ancestry	5642 Chinese ancestry	*MIPEP*
Meng W. [90]	9 October 2012	High myopia	187 European ancestry	1064 European ancestry	NA	NA	*CDH8*, *DHX15*, *SAMD5*, *LINC02434*, *PSMD10P2*, *KNG1*, *RNU6-1129P*, *GNPATP*, *RNU6-66P*, *RNU4-10P*, *LINC00603*, *SPATA22*, *ASPA* and 53 genes/loci
Shi Y. [91]	28 February 2013	High myopia	665 Han Chinese ancestry	960 Han Chinese ancestry	2128 Han Chinese ancestry	3683 Han Chinese ancestry	*SNTB1*, *PCDH1*, *VIPR2*
Khor C.C. [92]	9 August 2013	High myopia	1603 East Asian ancestry	3427 East Asian ancestry	1241 East Asian ancestry	3559 East Asian ancestry	*SNTB1*, *ZFHX1B*
Simpson C.L. [93]	18 September 2014	Myopia	3923 European ancestry	11,696 European ancestry	4331 European ancestry	4169 European ancestry	*LAMA2*, *GJD2*, *RBFOX1*
Pickrell J.K. [94]	16 May 2016	Myopia	106,086 European ancestry	85,757 European ancestry	NA	NA	*LAMA2*, *LRRC4C*, *GJD2*, *RDH5*, *PRSS56*, *ZMAT4*, *DLG2*, *PCCA-DT*, *NDUFA12P1*, *SHISA6*, *BMP3* and 41 genes/loci
Meguro A. [95]	16 May 2020	High myopia	1632 Japanese ancestry	1586 Japanese ancestry	881 East Asian ancestry	9946 East and Southeast Asian ancestry	*HIVEP3*, *NFASC*, *ZC3H11B*, *CNTN4-CNTN6*, *FRMD4B*, *LINC02418*, *GJD2*, *RASGRF1*, *AKAP13*

* Epub data or the earliest publication data; ** If the study reported more than 10 significant genes/loci, then only the name of top 10 genes/loci were shown in the table.

The association of the rs13382811 SNP in *ZFHX1B* with severe myopia was also identified [92]. A GWAS of high myopia, including Japanese and Chinese subjects in the primary cohort and Japanese, Chinese, Malay, and Indian subjects in the replication cohorts, identified 9 loci (Table 2) [95]. Our own study on Chinese subjects in Hong Kong confirmed *ZC3H11B* as a susceptibility gene for both high and extreme myopia, as well as *ZFHX1B* and *SNTB* as susceptibility genes for extreme myopia [96]. Moreover, the rs644242 SNP on the *PAX6* gene was associated with extreme myopia but not lower-grade myopia [97].

In summary, over a hundred SNPs have been identified for myopia throughout the past decade. However, their biological relevance and mechanisms remain unclear; no SNP has yet been shown to cause myopia directly.

#### 3.2.2. Association Studies on Refractive Error

In 2010, a GWAS on refractive errors that surveyed 4270 individuals from the twins UK cohort found that SNPs at the transcription start site of RASGRF were associated with refractive error. This association was replicated in six adult cohorts of European ancestry (Table 3) [98]. Moreover, RASGRF up-regulation was detected in myopic sclera under a guinea pig model, indicating that this gene may be involved in the scleral remodeling process of myopia development [99]. Another GWAS found *GJD2* to associate with refractive error [100]. A recent study showed that mutants in Exon 1 of *GJD2* homologs in zebrafish induced a refractive error, consistent with the results of the human GWAS [101].

In 2013, a genome-wide meta-analysis from the international Consortium for Refractive Error and Myopia (CREAM), including 37,382 European and 8376 Asian subjects, identified 24 SNPs associated with refractive errors (Table 3) [102]. Another meta-analysis of five European cohorts revealed refractive errors being associated with the *RBFOX1* gene [103]. In 2015, *APLP2* was identified as a myopia susceptibility gene [104].

One year later, CREAM reported a joint meta-analysis, which tested the interaction effects of SNPs and education on refractive error [104]. The meta-GWAS identified 25 loci associated with refractive error, including *LAMA2*, *GJD2*, *KCNQ5*, *FBN1*, and *TOX* (Table 3). Another meta-GWAS by CREAM identified 25 loci associated with refractive error (Table 3) [105]. In 2018, a GWAS of corneal and refractive astigmatism in a large-scale European cohort (UK Biobank) identified a shared role in myopia susceptibility loci [106]. Over 20% of central corneal thickness (CCT)-related loci near or within Mendelian disorder genes were identified to be associated with myopia [107].

#### 3.2.3. Genetic Association Analyses Focusing on Axial Length Elongation in Myopia

The minor C allele of rs4373767 on *ZC3H11B* demonstrated a protective effect against high myopia and elongated axial length [108]. A meta-analysis of GWASs found eight additional genes associated with axial length [109]. Moreover, a GWAS of South India identified *WNT7B* as a CCT-related locus [110]. The *WNT7B* SNPs were associated with CCT in Latinos [111]. In our cohort of Hong Kong Chinese subjects, we found that the *RSPO1* SNP rs12144790 and the *WNT7B* SNP rs10453441 are associated with axial length among Chinese children [112].

**Table 3:** Genome-wide association studies in refractive error and axial length.

First Author	Publication Date *	Discovery Stage	Replication Stage	Genes/Loci **
**Phenotype:** Refractive error				
Hysi P.G. [98]	14 September 2010	4270 European ancestry individuals	13,414 European ancestry individuals	*RASGRF1*
Solouki A.M. [100]	14 September 2010	5328 individuals predominantly European ancestry (>99%)	10,280 individuals predominantly European ancestry (>99%)	*GJD2-ACTC1*
Verhoeven V.J. [102]	12 February 2013	Stage 1: 37,382 European ancestry individuals; Stage 2: 8376 Asian ancestry individuals (meta-GWAS)		*CD55*, *PRSS56*, *CHRNG*, *CACNA1D*, *LAMA2*, *CHD7*, *TOX*, *ZMAT4*, *RORB*, *CYP26A1* and 16 genes/loci
Stambolian D. [103]	12 March 2013	7280 European ancestry individuals	19,673 European ancestry individuals	*RBFOX1*
Fan Q. [105]	30 March 2016	40,036 European and 10,315 Asian ancestry individuals (meta-GWAS)		*LAMA2*, *GJD2*, *KCNQ5*, *FBN1*, *TOX*, *DIS3L*, *FAM150B-ACP1*, *LINC00340*, *A2BP1*, *RDH5* and 15 genes/loci
Tedja M.S. [113]	28 May 2018	Stage 1: 44,192 European and 11,935 Asian ancestry individuals; Stage 2: 104,293 European ancestry individuals; Stage 3: j meta-GWAS	95,505 European ancestry individuals	*LAMA2*, *GJD2*, *KCNQ5*, *LRRC4C*, *RDH5*, *RBFOX1*, *SNORA51*, *PRSS56*, *SHISA6*, *ZMAT4* and 130 genes/loci
Hysi P.G. [19]	30 March 2020	508,855 European ancestry individuals	34,079 European ancestry individuals	*LAMA2*, *GJD2*, *KCNQ5*, *RBFOX1*, *LRRC4C*, *BLOC1S1-RDH5*, *TOX*, *PRSS56*, *ZMAT4*, *SHISA6* and 439 genes/loci
**Phenotype:** Axial length				
Fan Q. [108]	7 June 2012	1860 Chinese adults, 929 Chinese children, and 2155 Malay adults	NA	*ZC3H11B*
Cheng C.Y. [109]	8 August 2013	12,531 European ancestry individuals	8216 Asian ancestry individuals	*RSPO1*, *ZC3H11B*, *GJD2*, *C3orf26*, *LAMA2*, *ZNRF3*, *CD55*, *ALPPL2*, *MIP*
Miyake M. [111]	31 March 2015	3248 Japanese ancestry individuals	5383 Asian and 2690 Caucasian ancestry individuals	*WNT7B*

* Epub data or the earliest publication data; ** If the study reported more than 10 significant genes/loci, then only the name of top 10 genes/loci were shown in the table.

#### 3.2.4. The Role of Macular Thickness in Myopia

Macular thickness changes are associated with axial length elongation in myopia, glaucoma and age-related macular degeneration (AMD) [114,115,116]. An amount of 139 relevant genetic loci have been identified to be associated with macular thickness. Among them, *TSPAN10* and *RDH5* are highly expressed in the retina; they were significantly associated with myopia under a previous GWAS [94,117]. Yet another GWAS for myopic maculopathy revealed a susceptibility locus at rs11873439 in *CCDC102B* [118].

The biological functions of most variants from the GWAS remain unexplored. Variants in the coding regions can be investigated through gene-editing methods, e.g., zinc-finger nucleases or CRISPR-Cas9, in both cell and animal models. However, there have been no functional studies for non-coding variants. As such, none of the SNPs associated with myopia in the non-coding regions have been explored, leaving them open for future research. Lastly, candidate SNPs located within untranslated regions, DNA regulatory elements, and non-coding RNAs can be explored both in vitro and in vivo with the assistance of silico experiments based on non-coding genome databases, including GENCODE [119], OMIM [120], and ncRNA-eQTL [121].

## 4. Gene-Environment Interactions

Both gene-gene interactions and gene-environment interactions affect the occurrence and development of myopia. Conventional environmental factors include living environment, education level, and socioeconomic status. Recently, behavioral factors, such as near work and outdoor activities, have been shown to associate significantly with myopia development and progression, especially when leading to high or extreme myopia. These lifestyle factors also interact with susceptible genes.

### 4.1. Parental Myopia: Inherited Genes and Lifestyle

Myopia exhibits familial clustering [122]. In the 1958 British birth cohort, a higher risk of myopia was associated with low birth weight for gestational age, etc. [123]. Our study of Hong Kong Chinese schoolchildren shows that parental myopia confers a strong independent effect on childhood myopia [124]. The effect of parental myopia is stronger than that for the amount of near work and for time spent outdoors. One study proposed that a small genetic component of school-age myopia could be detected under low environmental variation conditions [18]. 

### 4.2. Interactions of Genetic Variants with Environmental, Educational, and Lifestyle Factors

A study surveying an urban and a rural adult Chinese cohort found that myopia was associated with an urbanized living environment, higher educational background, female sex, decreasing visual acuity (VA), and nuclear cataracts [125]. The pooled odds ratios (OR) for myopia indicated that each additional hour spent outdoors per week was associated with a 2% reduction in the odds of developing myopia [126]. Time spent outdoors among Australian schoolchildren in Sydney was negatively associated with incident myopia among both younger (age 6 at baseline) and older (age 12 at baseline) study cohorts [127]. Furthermore, a meta-analysis of Singaporean studies showed that three genetic loci exhibited strong associations with myopic refractive errors among individuals with higher education, while the association was only marginally significant among those with a lower secondary education at most [128]. Furthermore, reading activity could be used as an index for near work among youth populations [129]. A study in Israel suggested that learning styles involving intensive reading and near work activities could play a role in myopia development [130]. Lesser near work should therefore be considered under myopia prevention strategies.

Sequence variants in *APLP2*, a myopia susceptibility gene, have interactive effects with reading time in association with longitudinal changes in refractive errors [104]. A quantile regression analysis revealed heterogeneity of size effects in a non-uniform and non-linear manner [131]. In China, myopia prevalence was higher among children attending schools in urban areas than in the rural countryside [132]. Moreover, the interactions between optical and environmental factors are a complex part of myopia etiology. The refractive development of the eyes can be influenced by the local living environment and time spent in different environments [133], in addition to dietary and biochemical factors. Our own meta-analysis showed that lower 25 (OH) D is associated with an increased risk of myopia without genetic association, suggesting that 25 (OH) D level can be used as a proxy indicator for time outdoors [134].

In brief, the interactions between the genetic and environmental factors of myopia susceptibility are complex. How myopia pathogenesis is affected by these interactions, along with the underlying mechanisms of pathogenesis, remains to be investigated.

## 5. Prevention of Myopia Onset and Progression

Currently, effective treatments for myopia include both pharmacological and optical interventions. However, optimized protocols are still being vigorously researched.

### 5.1. Prevention of Myopia Onset

One clinical challenge in addressing myopia is determining when to start treatment, especially among children, as myopia development is unpredictable. For instance, environmental risk factors can reduce axial length elongation and myopia incidence [135]. Conversely, parental myopia is associated with a greater risk of early-onset myopia among children of almost all ethnic groups, including Asian, Hispanic, non-Hispanic whites, and African American children [136]. A recent pooled analysis found that individuals with family histories of strabismus and maternal smoking during pregnancy had a higher risk of moderate/high hyperopia than of low/moderate hyperopia [137]. Regarding children’s outdoor activities, policy interventions to promote increased time outdoors in schools across Taiwan were followed by increasingly low VA among schoolchildren [138]. For children growing up in Sydney, patterns of myopigenic activity by age, ethnicity, and gender showed that the children’s activity pattern become more myopigenic with age, with girls exhibiting a stronger pattern than boys, and East Asian children exhibiting a stronger pattern than European Caucasian children [139].

A Mendelian randomization study to assess the genetic predisposition of education on myopia revealed a causal role of educational attainment in refractive error [140]. Nevertheless, predicting refractive error and developing personalized myopia prevention strategies should be possible in the future through further genetic and environmental investigations [19,140]. Myopia development involves complicated gene-gene and gene-environmental interactions that display high heterogeneity, with non-uniform, non-linear, and non-additive effects [129]. Establishing polygenic risk scores (PRS) with acceptable accuracy still requires a large amount of genetic, environmental, and ophthalmic data [141]. Nevertheless, a trio-based study of early-onset high myopia using next-generation sequencing and whole-exome sequencing (WES) suggested that de novo mutations of the BSG gene may play a causative role in myopia and may therefore serve as genetic predictors [142].

### 5.2. Prevention of Myopia Progression

Promoting increased time outdoors during school hours, combined with therapeutic and optical treatments to slow down myopic progression, could help us gain control over the myopia epidemic [143]. Topical atropine has shown good clinical effects of slowing down myopia progression among children in randomized clinical trials [144]. Furthermore, 0.01% atropine has minimal side effects and has comparable efficacy in controlling myopia progression [145]. In our own Low-Concentration Atropine for Myopia Progression (LAMP) study, among the 0.05%, 0.025%, and 0.01% atropine eye drops tested, 0.05% atropine was the most effective in controlling spherical equivalent (SE) progression and AL elongation over a period of 1 year [146]. In our 2-year follow-up results, the efficacy of 0.05% atropine was observed to be double that of 0.01% atropine; it remained the optimal concentration among the studied concentrations in slowing myopia progression [147]. The optical approach includes bifocal/progressive spectacles, orthokeratology, defocus spectacles, and contact lenses. Myopia progression can thus be prevented through therapeutic treatments and lifestyle adjustments, given that the important prerequisite of being able to predict myopia development in children is fulfilled.

## 6. Polygenic Risk Scores: Prediction for Early Intervention

With the emerging safe and effective methods of controlling myopia progression by pharmacological and optical methods, the prediction of myopia progression becomes not as important as in the past. The increasing myopic rate in the younger population triggers a severe burden of high myopia development and related complications in the recent future. Systematic school-based screening, followed by clinical referral, is considered an effective approach to controlling school myopia. Nevertheless, identification of those with a high risk of progression is still needed for early prevention and intervention. A personalized approach to myopia prevention and control is needed.

Polygenic risk scores (PRSs) are indicators that can be used for disease risk stratification and prognostication among ophthalmic diseases, including primary open-angle glaucoma and AMD [148]. The scores can be used to predict myopia progression for application of early, safe, well-tolerated and effective methods, such as atropine, to prevent or limit progression. Harnessing the development of Big Data and machine learning approaches, a large dataset has been collected from the electronic health records of Chinese schoolchildren to improve myopia prognosis [149]. Furthermore, a recent meta-GWAS of refractive error estimated myopia heritability to be 18.4% and suggested possible improvements in the accuracy of myopia prediction [19]. Another meta-analysis investigating the association between PRS and the risk of myopia showed cycloplegic autorefraction to be a better indicator of myopia risk [150]. Lastly, a meta-analysis of 3 GWASs, including 711,984 individuals, evaluated PRSs in 1516 participants [150].

A study that screened the known variants associated with refractive error for non-additivity (dominance) showed that non-additive effects had negligible impacts on the accuracy of a PRS for refractive error derived by using genome-wide significant GWAS variants [141]. In another recent genetic association study of 54,006 individuals, PRSs derived from a GWAS for high myopia were predictive of high myopia, low myopia, and hyperopia [151]. For the Singapore Cohort of Risk factors for Myopia (SCORM) cohort study, an Asian PRS was developed to predict high myopia among Chinese children. The study found that adding the PRS to other clinical information improved the prediction of high myopia risk (AUC = 0.77) in teenagers, suggesting that the new Asian PRS could be used together with parental myopia and could improve its predictive performance in detecting children at risk of high myopia [152]. Lastly, our group investigated the associations with myopia progression and PRSs of SNPs in six genes—*ZC3H11B*, *ZFHX1B*, *KCNQ5*, *SNTB1*, *GJD2* and *MET*—among Chinese children in Hong Kong [153]. We found that SNPs in three genes—*ZFHX1B*, *KCNQ5*, and *GJD2*—were associated with faster myopia progression among Chinese children than other ethnic groups; in particular, Chinese children exhibiting the highest PRS defined by the three gene SNPs should be given appropriate intervention for myopia retardation [153].

Genetics interact with environmental determinants, such as education level and time spent outdoors. Higher education levels increased the prevalence of myopia among Chinese adults [154]. On the other hand, the interactive effect between genetic susceptibility and education has been shown by studies of two independent population-based European cohorts [155]. Notably, a study on Chinese schoolchildren showed that outdoor activity reduced myopic risk [11]. More time spent outdoors was also associated with less myopia and greater hyperopic mean refraction after adjusting for near work, parental myopia, and ethnicity [156]. Polygenic risk scores could improve the prediction of refractive error and the development of personalized myopia prevention strategies in the future.

## 7. Conclusions

Vigorous research worldwide has made substantial progress in exploring the myopic genetic basis over the last few decades. Linkage analyses have identified 26 myopia loci. NGS studies and familial congregation combined with functional analyses have identified putative pathogenic mutations in several genes. Given the diversity of myopia expressions in its environmental burdens and geographical patterns, as well as the diversity of patients’ backgrounds, more intriguing discoveries are warranted through the joint efforts of multiple research centers in the future. The associations of susceptibility genes with axial length, refractive error, and high myopia have been investigated for decades. However, genetic information, specifically on myopic maculopathy, is still limited and requires further investigation. More extensive studies are needed to examine the expanded spectrum of myopic mutations, genotype-phenotype correlations, novel gene identification, and biological characterization. Lastly, studies with animal models are expected to consolidate our understanding of myopia genetics.

## Figures and Tables

**Table 1:** Gene loci identified for myopia from linkage analysis and sequencing studies.

Locus	Location	Gene	Inheritance	Ethnicity	Reference
*MYP1*	Xq28	*OPN1LW*, *OPN1MW*	X-linked	Danish	Schwartz et al., 1990 [32]
*MYP2*	18p11.31	n.r.	AD	Chinese and European	Young 1998, Young 2001 [33,34]
*MYP3*	12q21-q23	n.r.	AD	German/Italian	Young 1998 [35]
*MYP5*	17q21-q22	n.r.	AD	English/Canadian	Paluru et al., 2003 [36]
*MYP6*	22q13.33	*SCO2*	AD	Ashkenazi Jewish	Stambolian et al., 2004 [37]
*MYP7*	11p13	n.r.	Multifactorial	Caucasian	Hammond et al., 2004 [38]
*MYP8*	3q26	n.r.	Multifactorial	Caucasian	Hammond et al., 2004 [38]
*MYP9*	4q12	n.r.	Multifactorial	Caucasian	Hammond et al., 2004 [38]
*MYP10*	8p23	n.r.	Multifactorial	Caucasian	Hammond et al., 2004 [38]
*MYP11*	4q22-q27	n.r.	AD	Chinese	Zhang et al., 2005 [39]
*MYP12*	2q37.1	n.r.	AD	Northern European	Paluru et al., 2005 [40]
*MYP13*	Xq23-q27.2	n.r.	X-linked	Chinese	Zhang et al., 2006 [41]
*MYP14*	1q36	n.r.	n.r.	Ashkenazi Jewish	Wojciechowski et al., 2006 [42]
*MYP15*	10q21.1	n.r.	AD	Caucasian	Nallasamy et al., 2007 [43]
*MYP16*	5p15.33-p15.2	n.r.	AD	Chinese	Lam et al., 2008 [24]
*MYP17*	7p15	n.r.	AD	French and Algerian	Paget et al., 2008 [44]
*MYP18*	14q22.1-q24.2	n.r.	AR	Chinese	Yang et al., 2009 [45]
*MYP19*	5p13.3-p15.1	n.r.	AD	Chinese	Ma et al., 2010 [46]
*MYP20*	13q12.12	n.r.	AD	Chinese	Shi et al., 2011 [47]
*MYP21*	1p22.2	*ZNF644*	AD	Chinese, Caucasian and African American	Shi et al., 2011 Tran-Viet et al., 2012 [48,49]
*MYP22*	4q35.1	*CCDC111*	AD	Chinese	Zhao et al. 2013 [50]
*MYP23*	4p16.3	*LRPAP1*	AR	Saudi Arabian and Chinese	Aldahmesh et al. 2013 Jiang et al. 2015 [51,52]
*MYP24*	12q13.3	*SLC39A5*	AD	Chinese	Guo et al., 2014 [53]
*MYP25*	5q31.1	*P4HA2*	AD	Chinese	Guo et al., 2015 [54]
*MYP26*	Xq13.1	*ARR3*	X-linked	Chinese	Xiao et al., 2016 [55]
*MYP27*	8q24.3	*CPSF1*	AD	Chinese	Ouyang et al., 2019 [56]

* *MYP4* was merged into *MYP17*. AD = autosomal dominant; AR = autosomal recessive; n.r. = not reported.
